# Functional promoter -765 G > C variant in COX-2 gene is associated with the susceptibility of breast cancer in Chinese Han women

**DOI:** 10.1186/1475-2867-14-38

**Published:** 2014-05-06

**Authors:** Jie Gao, Hua-Feng Kang, Xiao-Bin Ma, Wei Tang, Di Liu, Yang Zhao, Shu-Qun Zhang, Hai-Tao Guan, Shuai Lin, Hong-Tao Ren, Xi-Jing Wang, Zhi-Jun Dai

**Affiliations:** 1 Department of Oncology, the Second Affiliated Hospital of Xi’an Jiaotong University, Xi’an 710004, China; 2 Department of Nephrology, the Second Affiliated Hospital of Xi’an Jiaotong University, Xi’an 710004, China; 3 School of Chemistry and Chemical Engineering, Shaanxi Normal University, Xi’an 710061, Shaanxi Province, China

**Keywords:** Cyclooxygenase, COX-2, Polymorphism, Breast cancer, Susceptibility

## Abstract

**Background:**

Cyclooxygenase (*COX*) is a rate-limiting enzyme in prostaglandins synthesis which exists in two isoforms, COX-1 and COX-2. Over-expression of *COX-2* was considered to increase the proliferation and enhance the invasiveness of breast cancer cells. It was suggested that genetic variations in *COX-2* could influence its expression. Herein, the present study was aimed to investigate the associations between two mostly studied functional polymorphisms (-765 G > C and 8473 C > T) in *COX-2* and breast cancer risk in Chinese Han women.

**Methods:**

In the hospital-based case-control study, 465 breast cancer patients and 799 cancer-free controls were genotyped for the *COX-2* -765 G > C and 8473 C > T polymorphisms using TaqMan assay. We estimated odds ratios (ORs) and 95% confidence intervals (95% CIs) using the logistic regression.

**Results:**

Compared with the wild genotype of -765 G > C, we found a statistically significant increased risk of breast cancer associated with the variant genotypes [GC/CC *vs.* GG: OR = 1.56, 95% CI = 1.11–2.21]. In the stratified analysis, the increased risk was more predominant among the subgroups of younger subjects (OR = 1.61, 95% CI = 1.00–2.61). Furthermore, the variant genotypes were associated with large tumor size (OR = 3.01, 95% CI = 1.47–6.12). No significant association was observed for the 8473 C > T polymorphism.

**Conclusions:**

Our results suggest that the functional -765 G > C polymorphism in the promoter of *COX-2* may influence the susceptibility and progression of breast cancer in the Chinese Han population.

## Introduction

Breast cancer is the most common malignancy affecting women worldwide and its incidence rate is increasing in both developed and developing countries in recent years [1]. It is the second cause of cancer related mortality following lung cancer. The etiology of breast cancer has not been completely identified yet, but is thought to be multifactorial, with both environmental and genetic factors [2].

Cyclooxygenase is also known as prostaglandin endoperoxide synthetase which plays an important role in the inflammatory process through converting arachidonic acid to prostaglandins [3]. There are two isoforms of COX, COX-1 and COX-2. COX-1 is constitutive expressed and presents in many tissues while the inflammatory enzyme COX-2 is not detected in most normal tissues. However, COX-2 can be rapidly induced by a variety of mitogenic and inflammatory stimuli resulting in elevated levels of prostaglandins which can affect cell proliferation, apoptosis and angiogenesis, contributing to tumor occurrence and progression [4-6]. In breast cancer, several studies have suggested that moderate to high COX-2 expression is related to the genesis of mammary tumors and the expression is associated with parameters of aggressive breast cancer, including large tumor size, positive axillary lymph node metastases, and HER2-positive tumor status [7-10]. Targeted inhibition of COX-2 could inhibit the proliferation of breast cancer cell lines in vitro [11].

Genetic polymorphisms in *COX-2* have been shown to alter its expression and influence the susceptibility to various carcinomas [12-14], including breast cancer [15]. The human *COX-2* gene (also known as *PTGS2*) is located on chromosome 1q25.2-q25.3 and consists of 10 exons spanning 8.3 kb [16]. More than 15 single nucleotide polymorphisms (SNPs) in *COX-2* have been identified, but the most extensively studied polymorphisms are the -765 G > C (rs20417) in the promoter and the 8473 C > T (rs5275) in the 3′UTR of *COX-2*. Previous functional studies have suggested that the -765 G > C polymorphism may eliminate a Sp1-binding site but create an E2F binding site and result in altered *COX-2* expression [12,13]. The 8473 T > C polymorphism was shown to be associated with the alteration of mRNA level of the gene as sequences within the 3′UTR are important for message stability and translational efficiency [17]. There are also several studies that have investigated the association between the two SNPs and breast cancer risk; however the results from these studies are inconclusive. To make a more precise estimation, Yu et al. conducted a mete-analysis on the associations between several *COX-2* polymorphisms and breast cancer risk and suggested borderline significant increased risk of breast cancer was detected for rs5277 but no significant association was observed for the -765 G > C and 8473 C > T polymorphisms [15]. However, of the studies included in their meta-analysis, only two studies were conducted in Chinese population and none of the two studies investigated the rs5277 polymorphism since the minor allele frequency of rs5277 is very small in Chinese population [18,19]. Herein, in the present study, we investigated the association of the -765 G > C and 8473 C > T polymorphisms and breast cancer risk in a case-control study in a Chinese population.

## Materials and methods

### Study population

Our study consisted of 465 breast cancer patients which were consecutively recruited between May 2004 and September 2010 at the Second Affiliated Hospital of Xi’an Jiaotong University, China [20]. The Cases were recruited without the restriction of age. All of the patients were pathologically confirmed, sporadic breast cancer. Those patients that received chemotherapy or radiotherapy before surgery or had other type of cancer were excluded from the present study. For comparison, 799 cancer-free controls were recruited from subjects who were seeking health care in the outpatient departments at the hospital and were frequency-matched to the cases on age (±5 years). Before recruitment, a standard questionnaire was administered through face-to-face interviews by trained interviewers to obtain information on demographic data and related factors.

### Ethics statement

A consent was obtained from each study subject. The study protocol conformed to the Declaration of Helsinki and was approved by the ethics committee of the Second Affiliated Hospital of Xi’an Jiaotong University.

### Genotyping

The whole-genome DNA was isolated and purified from leucocytes of peripheral blood by proteinase K digestion and phenol/chloroform extraction, as described previously [20]. The two polymorphisms (-765 G > C and 8473 C > T) were genotyped by TaqMan MGB technology (Applied Biosystems, Foster City, CA, USA) according to the manufacturer’s instructions. The sequences of primer and probe for the two SNPs are available on request. The reaction mixture of 10 μL contained 20 ng genomic DNA, 3.5 μL of 2 × TaqMan Genotyping Master Mix, 0.25 μL of the primers and probes mix and 6.25 μL of double distilled water. The amplification was performed under the following conditions: 50°C for 2 min, 95°C for 10 min followed by 45 cycles of 95°C for 15 sec, and 60°C for 1 min. Amplifications were performed in the 384-well ABI 7900HT Real Time PCR System (Applied Biosystems), following the manufacturer’s instructions. After the completion of the amplification, the fluorescence intensity in each well of the plate was read and analyzed with SDS 2.4 automated software. Four blank controls were included in each plate to ensure accuracy of the genotyping. About 10% of the samples were randomly selected for repeated assays, and the results were in agreement with the results of the first assays.

### Statistical analyses

Differences in the distributions of demographic characteristics, selected variables, and frequencies distribution of genotypes the two SNPs between the cases and controls were evaluated using the Student’s t-test (for continuous variables) or χ^2^-test (for categorical variables). SNP allele frequencies in the controls were tested against departure from the Hardy-Weinberg equilibrium (HWE) using a goodness-of-fit χ^2^-test before analysis. The associations between the genotypes of the *COX-2* -765 G > C and 8473 C > T polymorphisms and risk of breast cancer and patients’ clinical characteristics were estimated by computing odds ratios (ORs) and 95% confidence intervals (CIs) from unconditional logistic regression analysis with the adjustment for age and age at menarche. *P* value less than 0.05 was considered statistically significant, and all statistical tests were two sided. All of the statistical analyses were performed with the software SAS 9.1.3 (SAS Institute, Cary, NC, USA).

## Results

### Characteristics of the patients and controls

The clinical information of the patients group and the demographic characteristics of both the cases and controls were present in Table 1. The cases and controls were well matched on age (*P* = 0.257). The frequency of individuals with age at menarche less than 14 was significantly higher in the case group (case vs. control: 29.3% vs. 22.9%, *P* = 0.012). The percent of patients with tumor size less than 2 cm, 2 to 5 cm and more than 5 cm are 27.1%, 66.7% and 6.2%, respectively.

**Table 1:** Distributions of select variables in breast cancer cases and cancer-free controls

**Variables**	**Case (N = 465), %**		**Control (N = 799), %**		***P*** *
Age at diagnosis or recruitment (year)					
Mean ± SD	52.0 ± 11.0		50.8 ± 13.2		0.110
<52	239	51.4	437	54.7	0.257
≥52	226	48.6	362	45.3	
Age at menarche (year)					
Mean ± SD	14.55 ± 1.84		14.89 ± 1.76		0.002
<14	136	29.3	183	22.9	0.012
≥14	329	70.7	616	77.1	
Tumor size					
Less than 2 cm	126	27.1			
2 to 5 cm	310	66.7			
More than 5 cm	29	6.2			
LN involvement					
Negative	251	54.0			
Positive	214	46.0			
ER					
Negative	162	34.8			
Positive	303	65.2			
PR					
Negative	209	44.9			
Positive	256	55.1			
HER-2					
Negative	309	66.4			
Positive	156	33.6			

ER, estrogen receptor; PR, progestin receptor; LN, lymph node; HER-2, human epidermal growth factor receptor 2; *T-test or two-sided χ^2^-test.

### Association between COX-2 polymorphisms and risk of breast cancer

The genotype and allele frequencies of the *COX-2* -765 G > C and 8473 C > T polymorphisms are shown in Table 2. The genotype frequencies of the two SNPs in controls both conformed to HWE (*P* = 0.203 and 0.117 for -765 G > C and 8473 C > T, respectively). The frequencies of the GG, GC and CC genotype of -765 G > C polymorphism among cases were significantly different from those among controls (*P* = 0.021). The difference in the frequencies distribution of G and C allele among case and controls were also significant (*P* = 0.005). Compared with individuals with *COX-2* -765 GG genotype, individual with GC or GC/CC genotypes both had a significantly increased susceptibility to breast cancer (OR = 1.55, 95% CI = 1.09–2.00 and OR = 1.56, 95% CI = 1.11–2.21, respectively). These results suggested that the *COX-2* -765 G > C polymorphism had effect on breast cancer occurrence. However, we did not observe significant associations between the *COX-2* 8473 C > T polymorphism and breast cancer risk in any comparison, as shown in Table 2.

**Table 2:** **Genotype and allele frequencies of the *COX-2 *** **polymorphisms among the cases and controls and the associations with risk the breast cancer**

**Polymorphisms**	**Case (N = 465)**		**Control (N = 799)**		***P*** *	**OR (95% CI)**^**†**^
	**N**	**%**	**N**	**%**		
-765 G > C						
GG	394	84.7	719	90.0	**0.021**	1.00 (reference)
GC	67	14.4	76	9.6		**1.55 (1.09–2.20)**
CC	4	0.9	4	0.4		1.88 (0.47–7.65)
GG	394	84.7	719	90.0	**0.005**	1.00 (reference)
GC + CC	71	15.3	80	10.0		**1.56 (1.11–2.21)**
C	75	8.0	84	5.2	**0.005**	
8473 C > T						
TT	299	64.3	515	64.5	0.209	1.00 (reference)
TC	132	28.4	244	30.5		0.93 (0.72–1.21)
CC	34	7.3	40	5.0		1.46 (0.90–2.37)
TT	299	64.3	515	64.5		1.00 (reference)
TC + CC	166	35.7	284	35.5	0.956	
C	200	21.5	324	20.3	0.462	

CI, confidence interval; OR, odds ratio; Compared with individuals with COX-2 -765 GG genotype, individual with GC or GC/CC genotypes both had a significantly increased susceptibility to breast cancer. *Two-sided χ^2^ test for the distributions of genotype and allele frequencies.

^†^Adjusted for age and age at menarche.

### Stratified analysis of the COX-2 -765 G > C polymorphism and breast cancer risk

We then evaluated the effect of the *COX-2* -765 G > C polymorphism on breast cancer stratified by age. As shown in Table 3, the risk effect of the -765 G > C variant genotypes (GC/CC) were more pronounced in younger subjects (*P* = 0.049, OR = 1.61, 95% CI = 1.00–2.61) rather than old subjects (*P* = 0.053, OR = 1.60, 95% CI = 0.96–2.68). The same analyses were also performed for the 8473 C > T polymorphism, however, no positive result was observed (data not shown).

**Table 3:** **Stratification analyses on age between the *COX-2*-765 G > C polymorphism and risk of breast cancer**

**The-765 G > C genotypes**	**Case (N = 465)**		**Control (N = 799)**		***P*** *	**OR (95% CI)**^**†**^
	**N**	**%**	**N**	**%**		
Age < 52						
GG	204	85.4	395	90.4	**0.049**	1.00 (reference)
GC + CC	35	14.6	42	9.6		**1.61 (1.00–2.61)**
Age ≥ 52						
GG	190	84.1	324	89.5	0.053	1.00 (reference)
GC + CC	36	15.9	38	10.5		1.60 (0.96–2.68)

CI, confidence interval; OR, odds ratio. The risk effect of the -765 G > C variant genotypes (GC/CC) were more pronounced in younger subjects rather than old subjects. *Two-sided χ^2^ test for the distributions of genotype frequencies. ^†^Adjusted for age and age at menarche.

### Association between the COX-2 polymorphisms and clinical parameters of breast cancer patients

To determine whether the *COX-2* polymorphisms have effect on the clinical features of breast cancer patients, we then analyzed the association between the *COX-2* polymorphisms and a series of clinicopathologic parameters, including tumor size, lymph node metastasis, and the statuses of ER, PR and Her-2. As shown in Table 4, we found the frequency of the variant genotypes of *COX-2* -765 G > C polymorphism was significant higher in patients with larger tumor size (>2 cm) (*P* = 0.006, OR = 3.01, 95% CI = 1.47–6.12).

**Table 4:** **The associations between the *COX-2*-765 G > C polymorphism and clinical characteristics of breast cancer patients**

**Variables**	**COX-2 -765 G > C genotypes**		***P*** *	**OR (95% CI)**^**†**^
	**GG (N, %)**	**GC/CC (N, %)**		
Tumor size				
Less than 2 cm	116 (92.1)	10 (7.9)	**0.006**	1.00 (reference)
More than 2 cm	278 (82.0)	61 (18.0)		**3.01 (1.47–6.12)**
LN involvement				
Negative	206 (82.1)	45 (17.9)	0.084	1.00 (reference)
Positive	188 (87.9)	26 (12.1)		0.63 (0.38–1.07)
ER				
Negative	140 (86.4)	22 (13.6)	0.459	1.00 (reference)
Positive	254 (83.8)	49 (16.2)		1.27 (0.62–2.61)
PR				
Negative	179 (85.7)	30 (14.3)	0.620	1.00 (reference)
Positive	215 (84.0)	41 (16.0)		0.97 (0.49–1.93)
HER-2				
Negative	262 (84.8)	47 (15.2)	0.961	1.00 (reference)
Positive	132 (84.6)	24 (15.4)		1.07 (0.61–1.86)

ER, estrogen receptor; PR, progestin receptor; LN, lymph node; HER-2, human epidermal growth factor receptor 2; CI, confidence interval; OR, odds ratio. The frequency of the variant genotypes of COX-2 -765 G > C polymorphism was significant higher in patients with larger tumor size (>2 cm). *Two-sided χ^2^ test for the distributions of genotype and allele frequencies. ^†^Adjusted for tumor size, LN involvement, ER, PR and HER-2 status.

## Discussion

In the present study, we found a significant association between the *COX-2* -765 G > C polymorphism and breast cancer risk. Compared with individuals with -765GG genotype, those with GC or GC/CC genotypes had a significantly increased breast cancer risk of 1.55 and 1.56, respectively. The risk effect was more predominant in younger individuals. Besides, we also found the *COX-2* -765GC/CC genotypes were associated with lager tumor size, suggesting the variant genotypes of this polymorphism may participate in the progression of breast cancer.

Over-expression of *COX-2* is observed in a variety of human malignancies and in premalignant lesions [21-23]. It is not only reported to be associated with the development of breast cancer but also plays important role in the progression of the disease. Previous functional studies suggested that the *COX-2* -765 G > C polymorphism was a functional variant and could alter *COX-2* expression, however, with inconclusive results [12,13]. For instance, Papafili et al. reported that -765C allele was associated with significantly reduced *COX-2* expression compared with the -765G allele and they postulated this effect to be mediated by the loss of Sp1 transcription factor binding to its cognate element [12]. However, Szczeklik et al. further demonstrated that -765C had a functional consequence resulting in enhanced biosynthesis of prostaglandins through eliminating a Sp1-binding site but creating an E2F-binding site [13]. Based on these findings, it would be interesting to investigate whether *the COX-2* -765 G > C polymorphism could be used as a genetic biomarker to predict cancer susceptibility and progression. In our study, we have observed that variant genotypes of the *COX-2* -765 G > C (GC/CC) were associated with increased breast cancer risk, and in patients group that were also associated larger tumor size. Our results are consistent with some studies on other malignancies. For example, Panguluri et al. reported that -765C allele was associated with an increased risk of prostate cancer in African Americans [24], and Koh et al. demonstrated that an elevated risk of colon cancer in a Singapore Chinese population [25]. There are also several studies on the association between the *COX-2* -765 G > C and breast cancer risk; however, the results are inconclusive. Recently, Yu et al. conducted a mete-analysis on this issue and concluded that no significant association was observed for the -765 G > C polymorphisms [15]. However, only two studies enrolled in the mete-analysis were conducted in Chinese population [18,19], which seems insufficient to make a conclusive result in the population. Besides, in consideration of the role of COX-2 in breast cancer, if the polymorphism is associated with increased COX-2 activity, the observed association in our study is biologically plausible. However, there is still need to investigate the functional effect of the *COX-2* -765 G > C polymorphism in breast cancer.

In the subgroup analysis, we found the increased risk associated with the *COX-2* -765 G > C polymorphism was more prominent in individuals younger than 52 years of age. As it is known, as age increases, individuals are more susceptible to many types of cancer, thus the small effect of the polymorphism may be overwhelmed. However, this observation should be interpreted with caution since we can not rule out the possibility that the number of older women in the older section have been decreased by deaths. We also observed that the *COX-2* -765 GC/CC genotypes was associated with larger tumor size. As we mentioned above, over-expression of COX-2 correlated with parameters of aggressive breast cancer, including large tumor size, positive axillary lymph node metastases, and HER2-positive tumor status. A better understanding of the function of the polymorphism in breast cancer may help to reveal the mechanism underlying the associations. However, the results should be interpreted cautiously since there is the possibility that the association may due to a late stage at diagnosis. Nevertheless, if confirmed by additional studies, this polymorphism may help to accurately predict the clinical course of breast cancer.

As our study was hospital-based design, we could not rule out the possible of selection bias of subjects that may have been associated with a particular genotype. However, the genotype distributions of the *COX-2* polymorphism in our study population were similar to distributions reported in other studies [18], and also similar to the distributions reported in the HapMap database for Han Chinese in Beijing [5.2% vs. 6.7% for -765 G > C and 20.3 vs. 19.5 for 8473 C > T]. Besides, the genotype distributions of the *COX-2* polymorphisms in our study population all conformed to HWE. Therefore, the selection bias in terms of genotype distribution would not be substantial.

In conclusion, our case-control study indicates that the *COX-2* -765 G > C polymorphism has a significant influence on the occurrence and progression of breast cancer in the Chinese population. Further functional studies are still required to investigate the role of -765 G > C variation in regulating the gene expression in breast cancer. Since our sample size is moderate and the statistical power of the study is limited, therefore, large population-based prospective studies are warranted to further elucidate the impact of the *COX-2* polymorphisms on breast cancer.

## Competing interest

The authors declare that they have no competing interests.

## Authors’ contributions

DZJ, GJ and KHF designed the research. DZJ, GJ, KHF, LD and MXB performed the experiments throughout this research. TW, ZY, ZSQ, GHT and WXJ contributed to the reagents, and participated in its design and coordination. LS and RHT analyzed the data; GJ, KHF and MXB contributed to the writing of the manuscript. Co-first authors: GJ, KHF, MXB and TW. All authors have read and approved the final manuscript.
